# Wavelet coherence analysis of cerebral oxygenation signals measured by near-infrared spectroscopy in sailors: an exploratory, experimental study

**DOI:** 10.1136/bmjopen-2016-013357

**Published:** 2016-11-03

**Authors:** Lingguo Bu, Jianfeng Li, Fangyi Li, Heshan Liu, Zengyong Li

**Affiliations:** 1 Key Laboratory of High Efficiency and Clean Mechanical Manufacture, School of Mechanical Engineering, Shandong University, Jinan, Shandong, P. R. China; 2 Key Laboratory of Rehabilitation Aids Technology and System of the Ministry of Civil Affairs, National Research Center for Rehabilitation Technical Aids, Beijing, P. R. China

**Keywords:** Near-infrared spectroscopy, Occupational health, Sailors, Wavelet phase coherence, Correlation analysis

## Abstract

**Objective:**

The objective of this study was to assess the effects of long-term offshore work on cerebral oxygenation oscillations in sailors based on the wavelet phase coherence (WPCO) of near-infrared spectroscopy (NIRS) signals.

**Methods:**

The fatigue severity scale (FSS) was first applied to assess the fatigue level of sailors and age-matched controls. Continuous recordings of NIRS signals were then obtained from the prefrontal lobes in 30 healthy sailors and 30 age-matched controls during the resting state. WPCO between the left and right prefrontal oscillations was analysed and Pearson correlation analysis was used to study the relationship between the FSS and the wavelet amplitude (WA), and between the FSS and the WPCO level.

**Results:**

The periodic oscillations of Delta (HbO_2_) signals were identified at six frequency intervals: I (0.6–2 Hz); II (0.145–0.6 Hz); III (0.052–0.145 Hz); IV (0.021–0.052 Hz); V (0.0095–0.021 Hz); and VI (0.005–0.0095 Hz). The WA in intervals I (F=8.823, p=0.004) and III (F=4.729, p=0.034) was significantly lower in sailors than that in the controls. The WPCO values of sailor group were significantly lower in intervals III (F=4.686, p=0.039), IV (F=4.864, p=0.036) and V (F=5.195, p=0.03) than those of the control group. In the sailor group, the WA in interval I (r=−0.799, p<0.01) and in interval III (r=−0.721, p<0.01) exhibited a negative correlation with the FSS. Also, the WPCO exhibited a negative correlation with the FSS in intervals III (r=−0.839, p<0.01), IV (r=−0.765, p<0.01) and V (r=−0.775, p<0.01) in the sailor group.

**Conclusions:**

The negative correlation between WA and FSS indicates that the lower oscillatory activities might contribute to the development of fatigue. The low WPCO in intervals III, IV and V represents a reduced phase synchronisation of myogenic, neurogenic and endothelial metabolic activities respectively and this may suggest a decline of cognitive function.

**Strengths and limitations of this study**

- Near-infrared spectroscopy (NIRS) is a suitable and easy-to-manage method for monitoring cerebral cortical oxygenation continuously and non-invasively at rest or during brain activation.
- This study provides a method for assessing the sailors' occupational risk such as fatigue and cognitive function decrease based on the wavelet phase coherence of NIRS signals.
- A limitation of this study is the small number of test samples. The generalisation of these findings still needs to be substantiated in the future.

## Introduction

With a long history, maritime transport involves a high amount of international trade in goods, especially for bulk material strategy, including oil and coal. As a result, maritime transport plays an irreplaceable role in global trade. As special occupational groups, ocean seafarers live in vile working and living environment on-board with noise and shock for a long period of time; moreover, they are separated from their families.^1^ The special occupational factors of sailors cause chronic fatigue, which might lead to a higher risk of subhealth.^2^ ^3^ The incidence of hypertension and hyperlipidaemia in ocean sailors is significantly higher than those on land.^4^

Recently, researchers have been interested in spontaneous oscillations in the cerebral haemodynamic signals for their possible roles in the monitoring of cerebrovascular pathology and functional activation. Spontaneous oscillations are considered to be related to vessel stiffness and a reduction of spontaneous low frequency oscillations is considered to indicate an increased vessel stiffness.^5^ Increased arterial stiffness is an independent risk factor for the development of atherosclerosis disease.^6^ However, there is little information about the spontaneous oscillations in sailors.

The fatigue of sailors leads to high risk of subhealth, which may be an early warning indicator of many diseases including cerebrovascular diseases. Therefore, it is important to devise methods to detect and quantify the fatigue level of sailors. Existing methods used to detect the fatigue of sailors mainly include the following ones: (1) subjective survey, such as surveys and diary studies;^7^ (2) behavioural survey, such as visual analogue mood scales, variable fore-period simple reaction time task, focused attention task and categorical search task;^8^ (3) objective survey, such as salivary cortisol and lactate test.^3^ Some progress has been made in domestic and foreign research on fatigue monitoring technology of sailors so far, however, there are still a number of problems in terms of validity and reliability. For example, the existing monitoring methods fail to well quantify the relationship between the sailor's fatigue and the monitoring index, and warning is not timely enough. Seeking a new simple and reliable real-time detection method is one of the hot spots in the research of fatigue monitoring for sailors.

Oxygen is one of the most important substances needed to maintain the physiological functions of human body. Low brain oxygenation may cause a mismatch between brain oxygen demand and oxygen supply, leading to reduced oxygen interstitial pressure and cellular pressure. The human brain can perform its functions only when the oxygen supply is sufficient.^9^ Near-infrared spectroscopy (NIRS) allows for non-invasive monitoring of regional changes in cerebral tissue oxygenation. NIRS is sensitive to the microvasculature and can measure changes in the concentration of oxyhaemoglobin (Delta (HbO_2_)).^10^ NIRS equipment emits the near-infrared light into the human tissue through the light source, and the absorption spectra of near-infrared light passing through the tissue are detected by the detector. The relative concentration of oxygenated haemoglobin (HbO_2_) in the region can be calculated by Beer-Lambert law. The spatial resolution of NIRS is lower than that of functional MRI (fMRI).^11^ Compared with methods including fMRI, NIRS has following advantages in the study of brain function:^12^ (1) Moderate temporal and spatial resolution, which makes it possible to avoid aliasing caused by the heart and respiration components in oxygen signal, as well as to detect the concentration changes of HbO_2_ and deoxygenated haemoglobin (HHb) in microcirculation of brain tissue; (2) portable, convenient, inexpensive and fewer constrains for subjects in experiment; (3) online real-time monitoring, and more suitable for brain functional detection of sailors. In recent years, NIRS has been used to evaluate changes in cerebral oxygenation and blood volume under various experimental conditions. For example, the changes in cerebral oxygen under the long-term simulated conditions can be evaluated by the NIRS method, and the fatigue of drivers can be assessed according to the changes in cerebral oxygen pulsation.^9^ Watanabe *et al*^13^ assessed the effects of creatine on mental fatigue revealed by cerebral HbO_2_ changes through NIRS method.

Spectral analysis of oxygenation signals deals with the dynamics of the tissue oxygenation and has been introduced as a method for the evaluation of regulation mechanism of cerebral and tissue vascular systems.^14^ ^15^ With spectral analysis based on wavelet transform of NIRS signal, several characteristic frequency intervals have been identified. Wavelet phase coherence (WPCO) reflects the synchronisation between the signals measured from two different brain regions, by assessing the level of time match between the two signals in time domain. This information of synchronisation could be used to evaluate the functional relationship of different cerebral cortical areas.^16^ However, little information is known on the dynamics of the cerebral tissue oxygenation and synchronisation in sailors.

In this study, we hypothesise that long-term offshore work might affect the cerebral oxygenation oscillation in sailors, and lead to a change in cerebral oxygenation dynamics as well as in the phase synchronisation in the left and right prefrontal regions. Wavelet analysis was used to deal with cerebral oxygenation signals and obtain the wavelet amplitude (WA) of the sailor and control groups. Changes in the phase coherence of prefrontal tissue Delta (HbO_2_) signals were assessed using WPCO method in sailor and control groups between left and right prefrontal lobes at rest. Then, the correlation analysis was conducted between the fatigue severity scale (FSS) and the WA, and between the FSS and the WPCO. This study would provide an insight into the fatigue development and cognitive function decrease.

## Materials and methods

### Subjects

A total of 30 sailors (age: 26.3±6.8 years) were recruited from ocean navigation. The sailors were training at Qingdao Ocean Shipping Mariners Company, Shandong Province off-board and recruited with pay. The average time off-board was <7 days and their average working years at sea was 7.3 years. A total of 30 age-matched controls (age: 26.1±6.4 years) were recruited from Shandong University. The subjects were recruited voluntarily, and all subjects were contacted in advance. All kinds of alcoholic drinks were not allowed within the 24 hours before the test in this study.

All subjects meet the following criteria: (1) no hypertension; (2) no diabetes; (3) no subarachnoid haemorrhage and other symptoms of stroke; (4) no disease in the heart, liver, lung, kidney, etc; (5) no smoking or drinking habits; and (6) no additional medical treatment.

The researchers introduced the basic information to the subjects (including research purpose, research process, time taken in the study, the potential risk and benefit for subjects), and obtained their agreement. All participants were required to rest well prior to the experiment without involving in any physical activity. The experimental procedures were approved by the Human Ethics Committee of Shandong University and were in accordance with the ethical standards specified by the Helsinki Declaration of 1975 (revised in 1983). The basic information of the experimental subjects can be seen in table 1.

**Table 1:** Basic information of the experimental subjects

Characteristic	Sailor group	Controls group	p for difference
Age (years)	26.3±6.8	26.1±6.4	0.828
Height (cm)	174.5±8.5	172.1±6.1	0.07
Weight (kg)	71.6±9.9	70.3±10.2	0.233
Body mass index (BMI)	23.5±1.4	23.7±1.3	0.451
Systolic blood pressure (mm Hg)	131.1±13.5	123.4±13	0.000**
Diastolic blood pressure (mm Hg)	70.2±2.5	69.2±4.2	0.678

Values are presented as means and SDs and percentages.

p Values for differences are calculated using t-test for means and SDs, and χ^2^ test for percentages.

**p<0.01.

### Measurements

#### Questionnaire

Self-reporting is a common method in evaluating fatigue. It can provide effective information on fatigue level subjectively. FSS was used to assess fatigue. Basically in the FSS, the subjects were required to answer a short questionnaire to assess the fatigue level.

The FSS consists of the following nine items: (1) My motivation is lower when I am fatigued. (2) Exercise brings on my fatigue. (3) I am easily fatigued. (4) Fatigue interferes with my physical functioning. (5) Fatigue causes frequent problems for me. (6) My fatigue prevents sustained physical functioning. (7) Fatigue interferes with carrying out certain duties and responsibilities. (8) Fatigue is among my three disabling symptoms. (9) Fatigue interferes with my work, family or social life. Each item was measured by a seven-point Likert type scale. The seven-scale marks are indicated by numbers 1–7, in which 1 represents completely disagreeable and 7 represents completely agreeable. The total scores were obtained by the sum of nine items. The FSS total score was averaged by the individual item responses.^17^

#### Near-infrared spectroscopy

NIRS is a non-invasive method to monitor concentration changes of tissue HbO_2_ and HHb. The majority of NIR light absorption results from the presence of haemoglobin in the small arterioles, capillaries and venules of the microcirculation.^18^ Spectral analysis of NIRS signals deals with the dynamics of the cerebral tissue oxygenation.^14^ In the present study, the same characteristic frequencies in cerebral NIRS signals were found as in blood flow signals as with Stefanovska *et al*^19^ and Shiogai *et al*.^20^ The oscillations found in NIRS signals may reflect similar origins as in blood flow signals. The oscillations in intervals I (0.6–2.0 Hz) and II (0.145–0.6 Hz) reflect the effects of cardiac and respiratory activities, respectively.^10^ ^20^ ^21^ The cerebral oscillations in interval III (0.052–0.145 Hz) were suggested to originate locally from intrinsic myogenic activity of smooth muscle cells in resistance vessels and this myogenic mechanism may be partly under autonomic control.^20^ Within the brain, interval IV (0.021–0.052 Hz) is closely regulated through tight neurovascular coupling and partial autonomic control.^22^

##### Near-infrared spectroscopy measurement

As prefrontal cortex has a strong correlation with cognitive behaviour, balance, personality and other advanced neural information processing functions in terms of functionality,^23^ NIRS measurements were performed on this area using the multichannel tissue oxygenation An Heng monitor (TH200, developed by Tsinghua University, China). This NIRS system has been widely used and verified to have high reliability.^24^ ^25^ All the subjects were asked to sustain the sitting position during the experiment. Each sensor of the TH-200 consisted of a two-wavelength light emitting diode (LED) and two PIN diodes. The LED component served as the source of emitted light at 760 and 850 nm, whereas the PIN diodes served as the detectors. The sampling frequency of the instrument is 10 Hz.

##### Experimental procedures

Before the experiment, the basic information including height, weight and medical history were recorded. Each subject was familiar with the experimental programme before the experiment. Subjects were tested in a house with the appropriate temperature and without noise. They were seated in comfortable posture during testing. Continuous recordings of NIRS signals were then obtained from the prefrontal lobes in 30 healthy sailors and 30 age-matched controls during the resting state. The NIRS recording time is 20 min.

### Wavelet-based coherence analysis

Wavelet-based coherence analysis was described in previous studies.^10^ ^19^ ^24^ Wavelet transform is a way to transform the time series from the time domain to the time–frequency domain. WA was averaged to indicate frequency specificity over time domain.^24^ In this study, the wavelet transform was calculated at a frequency interval of 0.005–2 Hz. The upper limit of 2 Hz was set to include the heart rate frequency, whereas the lower limit of 0.005 Hz was selected to include possible regulatory mechanisms of the tissue oxygenation signal.^20^ ^21^ WPCO can reveal the degree of coherence of the two signals by calculating the instantaneous phase. The WPCO value was calculated by the frequency domain amplitude of the instantaneous phase difference, which was averaged over time.^26^ First, two time series 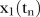 and 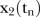 are obtained by the previous wavelet transform. Their corresponding instantaneous phases are 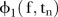 and 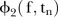 respectively. Second, calculate the instantaneous phase difference1
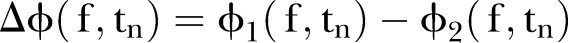


Third, 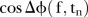 and 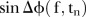 are averaged in the time domain. Finally, the phase coherence function is defined as^26^2
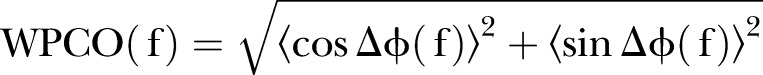


**Figure 1:**
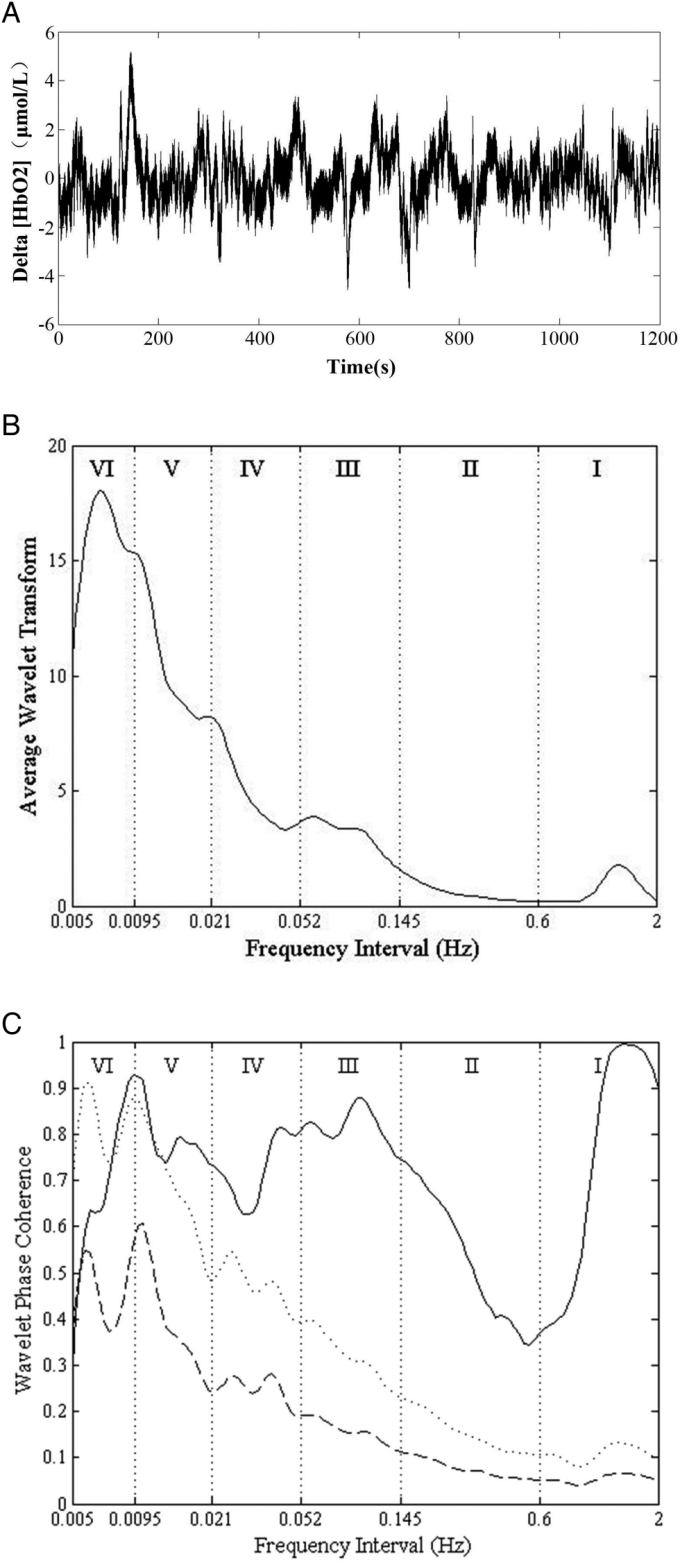
(A) The Delta (HbO_2_) signal, (B) the wavelet amplitude (WA). The vertical lines indicate the outer limits of the frequency intervals: (I, 0.6–2 Hz; II, 0.145–0.6 Hz; III, 0.052–0.145 Hz; IV, 0.021–0.052 Hz; V, 0.0095–0.021 Hz; and VI, 0.005–0.0095 Hz). (C) Wavelet phase coherence of two Delta (HbO_2_) signals. The solid line shows the wavelet phase coherence of two Delta (HbO_2_) signals. The dashed line and the dotted line show the mean and two SDs above the mean for the coherence calculated from 100 amplitude-adjusted Fourier transform (AAFT) surrogate signals, respectively.

It provides a new understanding of the dynamic adjustment of brain function from the angle of the phase. The range of phase coherence is between 0 and 1, which can be used to evaluate the phase coherence between the two signals at the same frequency and the same time. When the two vibration signals are not correlated, the phase coherence coefficient approaches to 0. The significant WPCO value was calculated as follows. First, the WPCO values were averaged across the subjects. Second, we used 100 amplitude-adjusted Fourier transform (AFFT) surrogate signals to calculate the mean surrogate WPCO value to evaluate the significance of the WPCO value in each frequency interval.^24^ ^27^ A WPCO value with two SDs above the mean surrogate was considered statistically significant. Third, the significant WPCO values were averaged across the interval. The average was performed with trapezoidal integral in each frequency intervals and divided it by the length of the frequency band. Figure 1 shows an example of the WA, WPCO of the left and right prefrontal Delta (HbO_2_) signals, mean and two SDs of AAFT surrogate signals.

### Correlation analysis

Correlation analysis is a statistical analysis method to study the linear relationship between the two variables. Pearson product–moment correlation coefficient is used to measure the correlation between the two variables (linear correlation) in statistics, and its value is between −1 and 1. In the field of natural science, this coefficient is widely used to measure the degree of correlation between the two variables. Karl Pierson modified this concept from a similar but slightly different idea presented by Francis Galton in 1880s. The correlation coefficient is also known as the Pearson correlation coefficient r. It is a value between 1 and −1, where ‘1’ indicates total positive correlation, ‘0’ indicates no correlation and ‘−1’ indicates total negative correlation.^28^

### Statistical analysis

All values were expressed as means and SDs. The data of the subjects were tested for normality (Kolmogorov-Smirnov test) and homogeneity of variance (Levene test) to ensure they meet the assumption required by the parameter analysis. One-way ANOVA was used to study the difference in the WA and WPCO of tissue oxygenation oscillations for each frequency band between the sailor and age-matched control subjects (SPSS V.11.5). A difference with p<0.05 was considered statistically significant.

## Results

### Fatigue level

**Figure 2:**
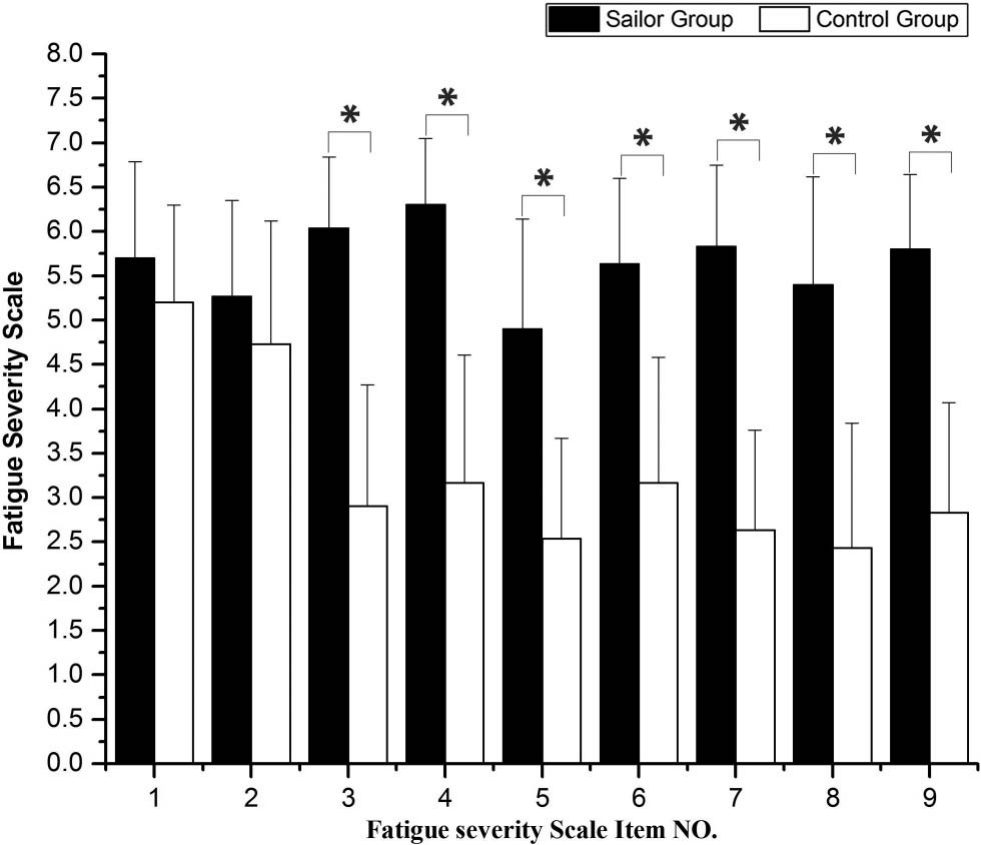
Comparison of fatigue severity scale (FSS) between the sailor group and control group. Significant differences are marked with *p<0.05 between the sailors and the age-matched controls.

Figure 2 shows the comparison of FSS between the sailor group and control group. Significant difference (p<0.05) in the scores from No. 3 to No. 9 was observed. The average score of subjective scale in 30 sailors was 5.65, and it was 3.29 in the age-matched control group. This indicates evident fatigue in the sailor group compared with the control group.

### Wavelet average amplitude

**Figure 3:**
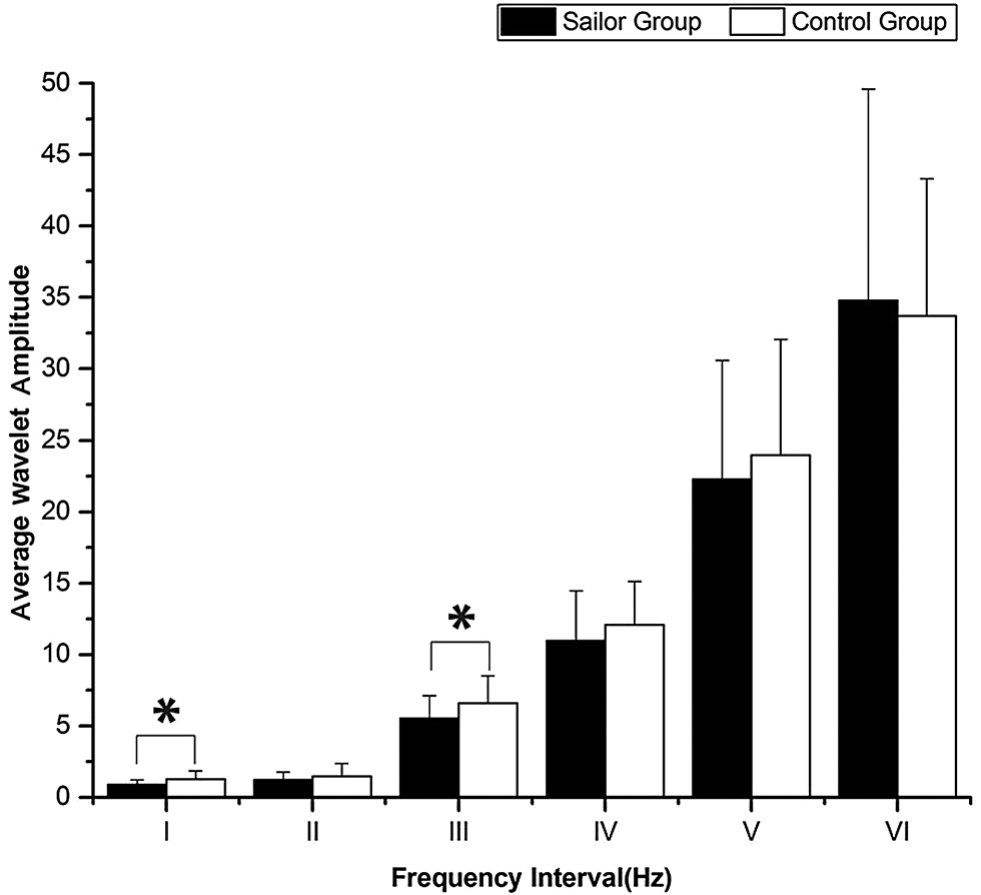
Comparison of the wavelet amplitude (WA) in the six frequency intervals between the sailors and the age-matched controls. Significant differences are marked with *p<0.05 between the sailors and the age-matched controls. Frequency intervals: I (0.6–2 Hz), II (0.145–0.6 Hz), III (0.052–0.145 Hz), IV (0.021–0.052 Hz), V (0.0095–0.021 Hz) and VI (0.005–0.0095 Hz).

The periodic oscillations of the Delta (HbO_2_) signals were identified at six frequency intervals: I, 0.6–2 Hz; II, 0.145–0.6 Hz; III, 0.052–0.145 Hz; IV, 0.021–00.052 Hz; V, 0.0095–0.021; and VI, 0.005–0.0095. Figure 3 shows the comparison of the WA of the age-matched controls and the sailors in the six frequency intervals. Significant difference was observed in frequency interval I and interval III between the sailors and age-matched controls (F=8.823, p=0.004 for interval I; F=4.729, p=0.034 for interval III).

The amplitude of the Delta (HbO_2_) oscillations in frequency interval I was found to be significantly lower by 15.1% in the sailors compared with that in the age-matched control subjects, and it was lower by 16.1% in interval III.

### Wavelet phase coherence

**Figure 4:**
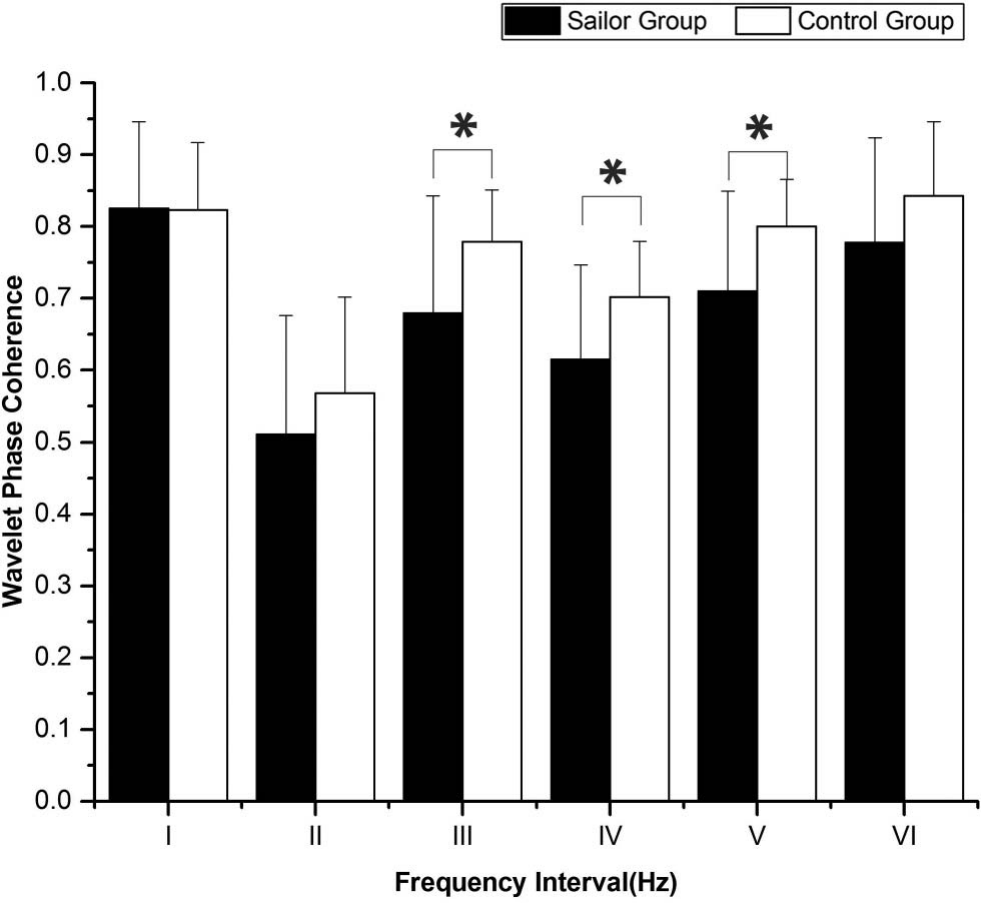
Comparison of the phase coherence in the six frequency intervals between the sailors and the age-matched controls. Significant differences are marked with *p<0.05 between the sailors and the age-matched controls. Frequency intervals: I (0.6–2 Hz), II (0.145–0.6 Hz), III (0.052–0.145 Hz), IV (0.021–0.052 Hz), V (0.0095–0.021 Hz) and VI (0.005–0.0095 Hz).

Figure 4 shows a comparison of the phase coherences of the left and right prefrontal Delta (HbO_2_) signals between the sailor and control groups. The phase coherences in intervals III, IV and V were significantly lower in sailor group than in control group (F=4.686, p=0.039 for interval III; F=4.864, p=0.036 for interval IV; F=5.195, p=0.03 for interval V). The WPCO value in frequency interval III was significantly lower by 12.72% in the sailors compared with that in the age-matched controls, while it was lower by 12.42% in interval IV and 11.30% in interval V.

### Relationship between fatigue level and WA and WPCO

Significant difference was observed in the average score (p<0.05) of FSS between the sailor group and control group. Nine items of FSS were averaged to obtain mean value of FSS in this study. The correlation analysis was carried out with this data.

**Figure 5:**
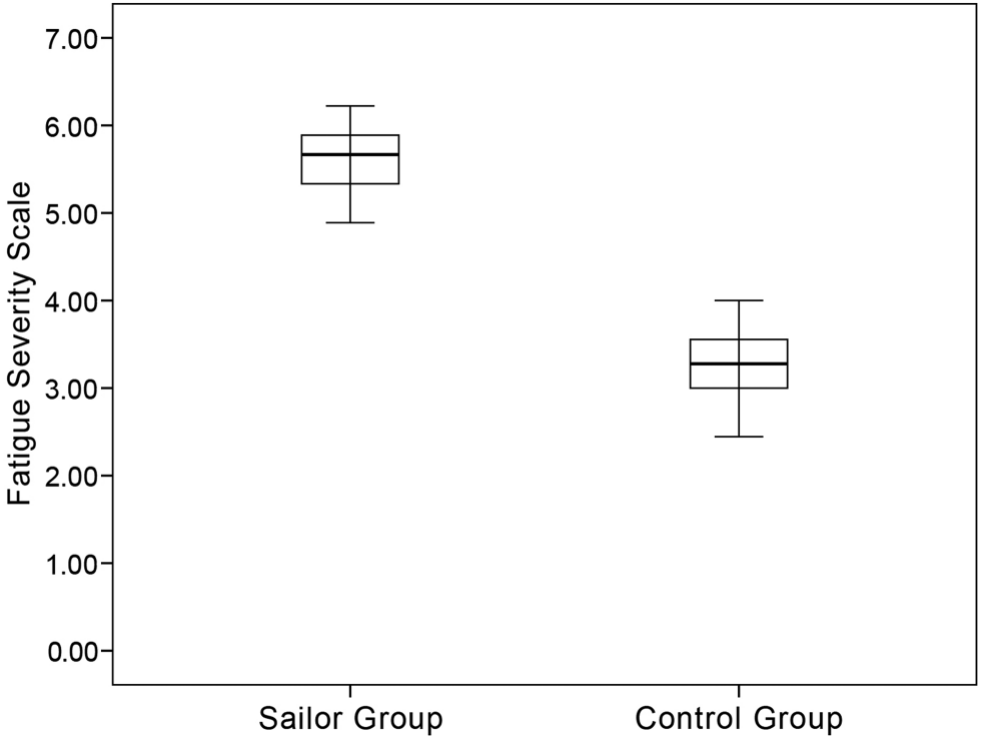
Boxplot is used to show the distribution of scores of subjective scale in sailor group and control group.

As shown in figure 5, boxplot was used to show the distributions of scores of sailor group and control group. Through one-way measures ANOVA, the score of subjective scale in sailors is significantly higher than that in controls (F=403.9, p<0.01).

**Figure 6:**
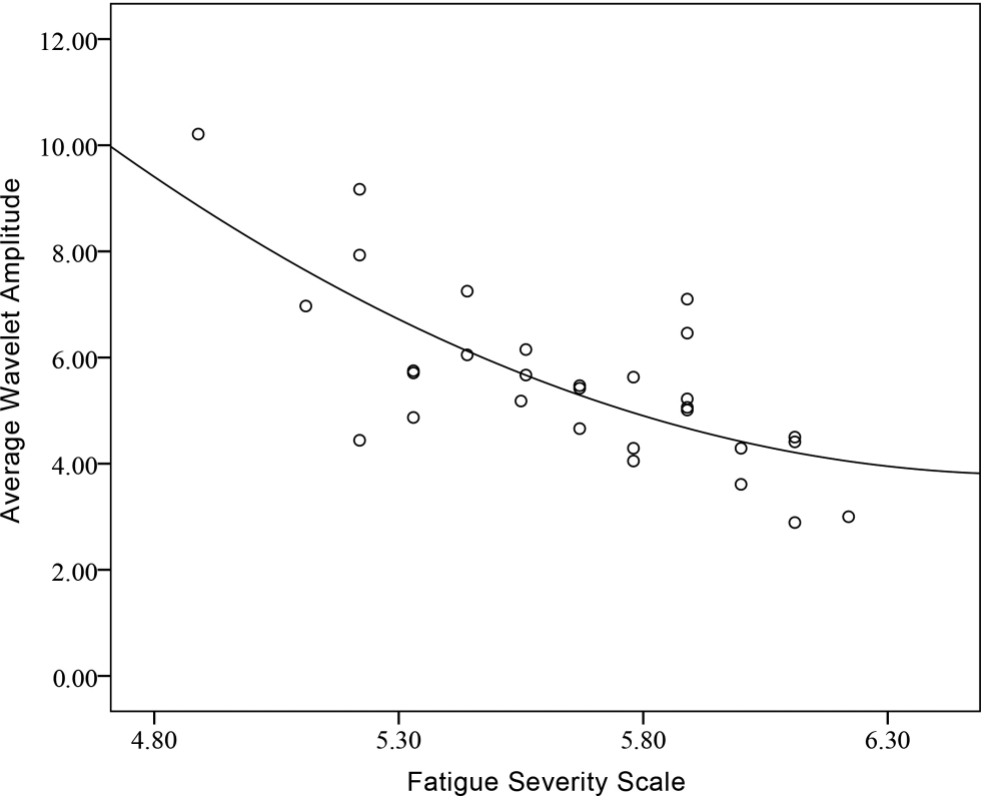
Correlation between fatigue severity scale (FSS) and wavelet amplitude (WA) in frequency interval III of sailor group.

In the sailor group, the correlation analysis showed that the correlation between FSS and WA in frequency interval I was very strong (r=−0.799, p<0.01). A negative correlation relationship was shown between the two. Similarly, the correlation analysis showed that the correlation between FSS and WA in frequency interval III was very strong in sailor group (r=−0.721, p<0.01). Figure 6 shows a negative correlation relationship between FSS and WA in frequency interval III in sailor group.

The correlation analysis showed that the negative correlation between FSS and WPCO values in frequency interval III (r=−0.839, p<0.01), interval IV (r=−0.765, p<0.01) and interval V (r=−0.775, p<0.01) was very strong in sailor group.

## Discussion

In the present study, the most important findings were as follows: (1) the WA in frequency intervals I and III in sailors was found to be significantly lower compared to that of the age-matched controls in resting state; (2) the WPCO values of sailor group were significantly lower in frequency intervals III, IV and V than those of the age-matched controls; and (3) the WA and the level of WPCO were found to be closely related to the fatigue level.

Cerebral oxygenation signal is a complicated signal that depends on several factors, such as blood flow, oxygen consumption, arterial saturation and arterial and venous volume.^29^ The oscillations in interval I reflect the effects of cardiac activity, which serves as a pump that drives blood through the vessels.^20^ As part of the systemic circulation, the cerebrovascular system is mediated by both central sympathetic activation and local myogenic or metabolic mechanisms.^30^ Hence, the significant lower oscillation of sailor group in interval I indicates a lower contribution of cardiac activity to the cerebral oscillations. The spectral amplitude of Delta (HbO_2_) in frequency interval III (0.052–0.145 Hz) was significantly lower in sailors compared with that in the age-matched controls. Frequency interval III is associated with changes in peripheral sympathetic nerve activity and reflects both sympathetically mediated and local myogenic mechanisms.^31^ Myogenic mechanisms buffer small changes in cerebral blood flow because of changes in systemic variables; the sympathetic nervous system is most active during large pressure changes.^32^ In the present study, the decreased amplitude in interval III might indicate a reduced contractility of the smooth muscle layer of the arteriolar in sailors. Oxygen is one of the most important substances to maintain the normal life activities of human body. Human fatigue is closely related to the supply of oxygen in cell metabolism, and hypoxia is an important cause of human fatigue.^33^ ^34^ Consequently, real-time assessment of the oxygenation status of human tissues under fatigue state is significant to reveal the mechanism of fatigue. Changes in the content of oxygen in the brain tissues when in motion are mainly caused by the changes of oxygen in the blood vessels. Thus, the changes in the content of HbO_2_ in vessels can indicate the changes in the content of oxygen in the brain.

Low phase coherence in intervals III, IV and V indicates a reduced connection between left and right prefrontal and this suggests a decline of cognitive function. The frequency interval III of spontaneous brain vibration (0.052–0.145 Hz) represents local intrinsic myogenic activity in smooth muscle cells, and the mechanism can be spontaneously adjusted and controlled.^24^ A low level of signal synchronisation is shown in the frequency interval III (0.052–0.145 Hz). Frequency interval III is caused by the myogenic activity of the local smooth muscle cells, and these activities can reflect the neural control circulation system of the brain. This suggests that long-term work in the sea may lead to the disorder of the coupling of the nerve activity in sailors, which leads to a reduction in the functional connectivity of the prefrontal lobe in the resting state.^25^ The frequency interval IV (0.021–0.052 Hz) mainly reflects the neurogenic activity. Sailors work for a long time under conditions such as high intensity of vibration and noise, which will lead to arterial dilation in some areas in brain, causing increased blood volume and blood flow. Therefore, a low phase coherence in the frequency interval IV indicates that neurogenic activity in the prefrontal region of the human body is activated under vibration conditions. In the frequency interval V (0.0095–0.021 Hz), it is mainly the endothelial metabolic activity to ensure that brain tissue gets enough oxygen from the blood. The normal blood flow was maintained in the brain vascular through regulation mechanisms of endothelial cell metabolism and local smooth muscle cell activity and regulation of neurogenic activity.^24^

There is a significant difference in systolic blood pressure between sailor group and control group after calculation. Cerebral perfusion pressure remains stable because the cerebral vasculature is continuously adapting to changes in arterial blood pressure.^35^ The relationship between cerebral blood flow and arterial pressure is considered to be a high-pass filter.^36^ Cerebral autoregulation is a frequency-dependent phenomenon, and the most effective operation frequency range is below 0.07 Hz.^36^ The low frequency component of blood pressure is effectively antiregulated, and the high frequency wave component is not affected by direct transmission.^31^ ^36^ In our study, the WA was significantly lower in intervals I and III in the sailors than that in controls. The WPCO values of sailor group were significantly lower in intervals III, IV and V than those of the control group. Therefore, these results might suggest that higher systolic blood pressure may partly contribute to the difference in WA and WPCO in the low frequency intervals between sailor and control group.

A significant negative correlation was observed between FSS and WA, as well as between FSS and WPCO, which proved that the fatigue level of sailors can be assessed, based on the WA and WPCO of NIRS signals.

Factors leading to fatigue in sailors include environmental, managemental and ship-specific factors.^3^ The spatial layout of the ship and long working hours on-board the ship would result in less exercise and overweight. Moreover, the usually unbalanced and high-fat diet aboard the ship in relation to long-time sailing could affect sailors' health. Moreover, entertainment on-board is less often, and sailors are under great pressure related to work. High stress load and isolation may cause psychosomatic disorders,^37^ such as fatigue. The mental stress caused a decrease of HHb in the bilateral prefrontal cortex.^38^ The FSS subjective assessment confirmed that the sailors easily get fatigued and experience frequent fatigue-related problems.

The generalisation of these findings still needs to be substantiated in the future. Future studies will focus on the effects of risk factors, such as vibration and noise on cerebral oxygenation oscillations.

## Conclusions

The cerebral oxygenation signals were detected and analysed based on the wavelet transform and WPCO of NIRS signals. The oscillations in intervals I and III were significantly lower in sailors than in age-matched controls. The phase coherences in intervals III, IV and V were significantly lower in sailor group than in control group. Long-term offshore work might lead to an insufficient supply of oxygen to the brain, thereby contributing to sailor fatigue. Low phase coherence indicates a reduced phase synchronisation between left and right prefrontal and this suggests a decline of cognitive function. This study would provide a method for assessing the sailors' occupational risk such as fatigue and cognitive function decrease.
